# Comparison of Inflammatory and Metabolic Markers During the Transition Period in Multiparous Holstein Cows with Cytological Endometritis, with or Without Concurrent Clinical Disease, and in Healthy Cows

**DOI:** 10.3390/ani16213445

**Published:** 2026-10-31

**Authors:** Liisa Siimon, Tanel Kaart, Merle Valdmann, Jevgeni Kurykin, Andres Waldmann

**Affiliations:** 1 Institute of Veterinary Medicine and Animal Sciences, Estonian University of Life Sciences, 51006 Tartu, Estonia; 2 Faculty of Veterinary Medicine, Latvia University of Life Sciences and Technologies, LV-3004 Jelgava, Latvia

**Keywords:** cytological endometritis, acute phase proteins, metabolic markers, transition period

## Abstract

**Simple Summary:**

Inflammatory uterine disease is a major cause of subfertility/infertility in postpartum dairy cows, resulting in substantial economic losses. At approximately eight weeks after calving, around 30% of cows experience uterine inflammation characterised by an increased proportion of white blood cells in the uterine lumen—a condition termed cytological endometritis. There is evidence that dysregulated systemic inflammation around calving predisposes cows to the development of cytological endometritis. As clinical diseases are also common in dairy herds, we examined changes in blood markers associated with inflammation before and after calving in multiparous Holstein cows that developed cytological endometritis, with or without concurrent clinical disease or calving-related disorder, and compared them with healthy cows. We found that the pattern of selected markers associated with the development of cytological endometritis differed according to the presence of concurrent disease or calving-related disorder. Compared with healthy cows, cows that developed cytological endometritis and had concurrent disease or disorder showed alterations in markers associated with inflammatory response already before calving and also at three weeks after calving. Changes in two inflammatory markers, haptoglobin at 7 days and albumin at 21 days postpartum, were associated with the development of cytological endometritis irrespective of concurrent disease or disorder and may help improve the identification of cows at increased risk before the condition is diagnosed, potentially facilitating earlier intervention.

**Abstract:**

Inflammation is a major risk factor for cytological endometritis (CYTO) in dairy cows. This study evaluated plasma concentrations of positive acute phase proteins (APPs) including haptoglobin, serum amyloid A and ceruloplasmin, as well as the negative acute phase protein albumin and the metabolic markers cholesterol and insulin-like growth factor-1 during the transition period. Blood was collected from multiparous Holstein cows at −14 days prepartum and 7 and 21 days postpartum. Endometrial cytology samples were collected at 40 ± 2 days postpartum, and CYTO was diagnosed using a polymorphonuclear neutrophil threshold of >8%. Cows were classified according to CYTO status and the presence of clinical disease or calving-related disorder (DIS) as CYTO/DIS− (*n* = 14), CYTO/DIS+ (*n* = 22) or HEALTHY (*n* = 50). APP and metabolic marker profiles associated with CYTO differed according to concurrent disease/disorder status. Plasma haptoglobin concentrations at 7 days postpartum were higher and albumin concentrations at 21 days postpartum were lower in both CYTO groups than in healthy cows (*p* < 0.05), suggesting that these markers may have potential value for identifying cows at risk of CYTO, irrespective of concurrent clinical disease or calving-related disorder. Further studies are needed to validate their predictive performance.

## 1. Introduction

The transition period, typically defined as the three weeks before to the three weeks after parturition, is a critical phase in the life of dairy cows. Current evidence suggests that the majority of dairy cows undergo a state of negative energy balance, adipose tissue mobilisation, immune activation and low-grade systemic inflammation during the transition period—even in the absence of overt metabolic or clinical disease [1,2]. A well-regulated postpartum endometrial inflammatory response is considered essential for bacterial clearance and re-establishment of endometrial function, thereby supporting a successful transition [3,4]. However, dysregulated inflammation—either insufficient or excessive—within the reproductive tract or at the systemic level may lead to the development of uterine inflammatory diseases [5,6,7].

Among uterine inflammatory diseases, cytological endometritis (CYTO) is commonly defined by the percentage of polymorphonuclear neutrophils (PMNs) exceeding a threshold (typically > 5%) in the uterine lumen, measured between 3 and 9 weeks postpartum [8,9,10,11]. CYTO is a prevalent postpartum disorder that adversely affects fertility and increases the risk of involuntary culling [7,8,12]. It occurs in both clinically healthy cows and cows affected by clinical diseases, with an approximately two-fold higher prevalence in diseased animals [12]. Importantly, recent evidence suggests that CYTO is an important determinant of reproductive performance, irrespective of concurrent clinical disease. Cows with CYTO exhibit lower pregnancy rates and an increased risk of culling, even in the absence of clinical disease. These adverse effects may be further exacerbated when CYTO co-occurs with clinical disease [11,12].

Acute phase proteins (APPs) are widely recognised as valuable biomarkers of inflammation, infection and tissue damage, and may also be useful in prognostication and detection of subclinical disease [13]. APPs are typically classified as positive (e.g., haptoglobin (Hp), serum amyloid A (SAA) and ceruloplasmin (CERULO)) and negative (e.g., albumin (ALB)) acute phase proteins, depending on whether their concentration increases or decreases during the acute phase response as a part of the innate immune response [13,14]. Positive APPs typically increase in concentration in response to infection or inflammation, whereas negative APPs decrease, as a result of altered hepatic protein synthesis [13,15,16]. While cholesterol (CHOL) is used as an indicator of lipid metabolism [17,18], some studies in dairy cows have also classified it as a negative acute phase metabolite [19,20], and decreased postpartum cholesterol concentrations have been reported to be associated with the incidence of metritis and the occurrence of multiple postpartum disorders [21]. Although insulin-like growth factor-1 (IGF-1) is commonly used as a metabolic and endocrine marker, it may also reflect inflammatory status. For example, in a sheep model, experimental induction of systemic inflammation by lipopolysaccharide has been shown to increase circulating growth hormone (GH) concentrations while reducing hepatic GH receptor expression, resulting in inflammation-induced GH resistance and decreased IGF-1 expression [22].

Subclinical CYTO has been proposed to represent a manifestation of inflammatory dysregulation [23], which may be reflected in alterations in APP concentrations. Changes in APPs during the postpartum period have been associated with the development of subclinical CYTO [23,24], as well as with other uterine disorders, including clinical endometritis [25,26], metritis [27], and retained placenta [28], and non-uterine disorders, such as lameness [29,30], ketosis [31] and subclinical hypocalcaemia [32]. However, given the high prevalence of postpartum clinical diseases (42–61%) in dairy herds [33,34], it remains unclear whether specific APP and metabolic marker profiles are associated with the development of CYTO independently of concurrent clinical disease.

To date, no studies have directly compared the temporal dynamics of multiple APPs and metabolic markers during the transition period in cows that develop CYTO with and without concurrent clinical disease or calving-related disorder, and in healthy cows. A better understanding of APP and metabolic marker patterns may provide insight into the mechanisms linking transition period physiology to the development of CYTO, and facilitate the identification of robust biomarkers for early risk stratification.

Therefore, the aim of the present study was to characterise the concentrations and temporal patterns of selected APPs and metabolic markers during the transition period in cows that developed CYTO with or without concurrent clinical disease or calving-related disorder, and to compare these patterns with those of CYTO-negative healthy cows.

## 2. Materials and Methods

### 2.1. Animals, Housing and Management

This observational study utilised the same dataset from earlier studies by our group [12,35]. The studies were approved by the Animal Experimentation Project Permit Commission at the Ministry of Rural Affairs, Estonia (13.05.2008 no 7.2-11/3240-1).

The study was conducted on a commercial dairy farm with approximately 1200 cows housed in a loose-housing system. All consecutively calving multiparous cows were enrolled in three experiments (Experiments 1–3). Cows culled before endometrial cytological sampling were excluded from the study. Experiments 1, 2 and 3 comprised 45, 64 and 10 cows, respectively. The sampling period for each experiment did not exceed 3.3 months; however, owing to intervals between experiments, the overall study period extended over 19 months.

For the present study, data from 86 multiparous Holstein cows were selected from the original dataset of 119 cows. The cows were enrolled three weeks before their expected calving date and were followed to at least day 65 of pregnancy or culling from the herd.

The cows were classified into three groups according to the presence of CYTO at 40 ± 2 days postpartum and the occurrence of clinical diseases or calving-related disorders. Cows without CYTO or any recorded calving-related disorder or clinical disease were classified as healthy (HEALTHY; *n* = 50). Cows diagnosed with CYTO but without any recorded clinical disease or calving-related disorder were assigned to the CYTO/DIS− group (*n* = 14). Cows diagnosed with CYTO and at least one calving-related disorder or clinical disease were assigned to the CYTO/DIS+ group (*n* = 22). Cows with calving-related disorder or clinical disease but without CYTO (*n* = 33) were excluded from the present analysis.

Cows were fed their respective diets in the form of a total mixed ration, administered twice daily to allow ad libitum consumption. Milking was conducted twice per day in a parallel rotary milking parlour (PER-40, DeLaval, Tumba, Sweden).

Daily milk yields were measured with flow milk meters (MM25, DeLaval, Tumba, Sweden), and the 45-day cumulative milk yield was calculated as the sum of the daily milk yields.

The clinical health status of the experimental cows was routinely monitored throughout the study period by the farm veterinarian during blood sampling at 14 days prepartum and 7 and 21 days postpartum, by farm personnel during twice-weekly milk sampling, and by a veterinary PhD student during body condition and locomotion scoring, which was performed every two weeks. Any abnormal clinical signs observed were reported to the farm veterinarian, who examined the affected cows, established a diagnosis and provided treatment when necessary. The calving-related disorders and clinical diseases diagnosed were twinning, dystocia, retained placenta, metritis, purulent vaginal discharge after 21 days in milk, clinical hypocalcaemia, ketosis, clinical mastitis and severe lameness.

Dystocia was defined as providing assistance without or with the use of mechanical traction. Retention of foetal membranes was characterised as the presence of foetal membranes at least 12 h after calving. Clinical hypocalcaemia was identified from the resolution of hypocalcaemia symptoms (incoordination when walking, decrease in body temperature and downer cow syndrome) after infusion of 10 g of calcium as calcium borogluconate. Ketosis was determined from reduced feed intake and/or milk yield and milk testing positive for ketone bodies (Ketostix strip; Bayer, Leverkusen, Germany). Clinical mastitis was characterised as the presence of abnormal milk or signs of inflammation in one or more quarters. All cows were examined for signs of clinical mastitis before each milking.

Metritis and PVD were defined according to Sheldon et al. [9]. At the time of blood sampling, 7 and 21 days postpartum, the farm veterinarian collected vaginal mucus samples using an EndoControl Sampler (REF: 17075/0020; Minitübe, Tiefenbach, Germany), and investigated the status of the reproductive organs by rectal palpation. Metritis was characterised by an abnormally enlarged uterus accompanied by a watery, reddish-brown or purulent uterine discharge, which could be fetid. Purulent vaginal discharge was defined as the presence of purulent (>50% pus) or mucopurulent discharge in the vagina at 21 days or more postpartum, without systemic signs of illness. All cows underwent one additional examination of vaginal mucus characteristics between 21 and 40 days postpartum.

The assessment of subclinical diseases during the postpartum period included subclinical ketosis and subclinical mastitis. Subclinical ketosis was assessed by measuring plasma β-hydroxybutyrate (BHB) concentrations at 7 and 21 days postpartum. BHB concentrations were determined according to Harano et al. [36] using an ADVIA 1650^®^ Chemistry System autoanalyser (Siemens Medical Solutions, Tarrytown, NY, USA). Subclinical ketosis was defined as plasma BHB concentrations ≥ 1.2 mmol/L [37]. Subclinical mastitis was assessed using somatic cell count (SCC) data from milk samples collected at 19.1 ± 9.7 days postpartum (mean ± SD) and was defined as an SCC exceeding 200,000 cells/mL [38]. SCC data were obtained from Estonian Livestock Performance Recording Ltd. (Tartu, Estonia). Subclinical hypocalcaemia was not evaluated in the present study.

Locomotion was scored every two weeks using the 5-point ordinal scale described by Sprecher et al. [39]. Cows with a locomotion score of 4 or higher were classified as severely lame and considered to meet the lameness criterion for clinical disease in the present study.

Body condition was scored according to Ferguson et al. [40] at week −2 prepartum using a 1 (emaciated) to 5 (obese) scale with 0.25-unit increments.

### 2.2. Blood Sampling

An experienced farm veterinarian collected blood samples from the coccygeal vein or artery into heparinised tubes (Terumo Europe, Leuven, Belgium) at three timepoints: −14 days prepartum, 7 days postpartum and 21 days postpartum. The actual times (mean ± SD) of the samplings were: −15.10 ± 5.10 days prepartum, 7.03 ± 1.05 days postpartum and 20.48 ± 1.05 days postpartum. Plasma was harvested within 30 min of blood collection by centrifugation at 900× *g* for 15 min. Samples were initially stored at −20 °C on the farm for one week, and thereafter at −80 °C until analysis.

### 2.3. Blood Analyses

IGF-1 concentrations were analysed using OCTEIA kits (Immunodiagnostic Systems, Tyne & Wear, Boldon, UK) as described by Swali and Wathes [41]. Prior to analysis, the samples were incubated with a reagent to inactivate binding proteins and then diluted for the assay. The kit measures total IGF-1 concentration with a detection limit of 3.1 ng/mL. SAA concentrations were measured by multispecies sandwich ELISA (Phase SAA kit, Tridelta Development Ltd., Maynooth, Ireland); the minimum detectable concentration of SAA is 0.3 ng/mL. Optical densities were read by a Sunrise™ microplate reader (Tecan, Grödig, Austria) and the results calculated using cubic spline regression with Magellan™ data analysis software V 7.2 (Tecan). The inter- and intra-assay coefficients of variation were <10% for both assays.

Plasma concentrations of Hp, CERULO, ALB and CHOL were measured at the Department of Animal and Veterinary Sciences, Aarhus University, Denmark, by our collaborator Dr. Torben Larsen and his staff. Plasma CHOL and ALB were determined according to standard procedures (Siemens Diagnostics^®^ Tarrytown, NY, USA Clinical Methods for ADVIA 1650). Hp concentration was determined chemically based on its ability to bind haemoglobin (Phase™, Tridelta Development Ltd., Wicklow, Ireland). CERULO activity was analysed according to Richterich [42]. All analyses were performed using an ADVIA 1650^®^ Chemistry System autoanalyser (Siemens Medical Solutions, Tarrytown, NY, USA). The intra- and inter-assay coefficients of variation were <4.0% and 5.1%, respectively, for all analyses.

### 2.4. Endometrial Sampling and Cyto Diagnostics

We have previously described these procedures in detail [35]. Briefly, endometrial samples for cytological evaluation were collected at 40 ± 2 days postpartum from the base of the presumed larger uterine horn using an endometrial brush (Uterobrush^®^, Medscand Medical, Malmö, Sweden) mounted on a stainless steel instrument designed for use in cattle [43]. All cytological sampling was performed by the same researcher. Smears were prepared by gently rolling the Uterobrush onto a clean microscope slide, followed by immediate fixation using warm air from a blow dryer. Staining was conducted using the May–Grünwald–Giemsa technique. Two slides per cow were examined at 400× magnification. Epithelial cells and PMNs were counted on each slide from a total of 100 cells, and the proportion of PMNs was calculated according to the method described by Kasimanickam et al. [8]. The mean PMN percentage from the two slides was used for further analysis. All cytological assessments were performed by the same evaluator. The diagnostic threshold for CYTO was set at >8% PMNs. This was the PMN percentage that yielded the highest combined sensitivity and specificity for predicting pregnancy at 180 days postpartum [8,12].

### 2.5. Grouping of Cows by Presence of CYTO and Health Status

In order to compare APP and metabolic marker concentrations during the transition period, cows were classified into groups based on CYTO status and the presence or absence of calving-related disorders or clinical diseases (DIS): CYTO/DIS− (*n* = 14), CYTO/DIS+ (*n* = 22) and HEALTHY (*n* = 50).

### 2.6. Statistical Analyses

The differences between groups (CYTO/DIS−, CYTO/DIS+, HEALTHY) in parity, body condition score (BCS) before calving, 45-day cumulative milk yield and uterine PMN % at 40 ± 2 days postpartum were tested with the Kruskal–Wallis test. The pairwise comparison of groups was performed with the pairwise Wilcoxon test, followed by multiple testing correction with the FDR method.

The effects of group and time period (−14 days prepartum, 7 days postpartum, 21 days postpartum) on studied blood metabolites were estimated with linear mixed models considering fixed effects of time, group, time by group interaction, parity (2nd vs. >2) and BCS before calving, and random effects of experiment and cow; right-skewed Hp and SAA were tested on a binary logarithmic scale. For pairwise comparison of groups within time periods and overall, as well as for comparison of time periods within groups and overall, the Tukey post hoc test was used.

Results were considered statistically significant at *p* ≤ 0.05. All statistical analyses were conducted in R (version 4.4.1, R Foundation for Statistical Computing, Vienna, Austria). For statistical modelling and hypothesis testing, the packages Ime4 [44], car [45] and emmeans [46] were used.

## 3. Results

### 3.1. Description of the Study Groups

Descriptive characteristics of the study groups are presented in Table 1. Parity differed between groups (*p* = 0.004), with CYTO/DIS+ cows having a higher parity than HEALTHY cows, while CYTO/DIS− cows showed an intermediate value that did not differ significantly from either group. Pre-calving BCS did not differ among the groups (*p* = 0.273). The 45-day cumulative milk yield was lower in CYTO/DIS+ cows compared with both HEALTHY and CYTO/DIS− cows (both *p* < 0.05), whereas no difference was observed between HEALTHY and CYTO/DIS− cows. Uterine PMN % at 40 ± 2 days postpartum was markedly higher in both CYTO groups compared with HEALTHY cows (both *p* < 0.001), with no significant difference between CYTO/DIS− and CYTO/DIS+ cows.

Calving-related disorders and clinical diseases diagnosed in the 22 cows in the CYTO/DIS+ group are presented in Table 2.

At 7 days postpartum, BHB concentrations exceeded 1.2 mmol/L in 7 of 50 cows (14.0%) in HEALTHY group, 2 of 14 cows (14.3%) in the CYTO/DIS- group, and 3 of 22 cows (13.6%) in the CYTO/DIS+ group. At 21 days postpartum, BHB concentrations exceeded 1.2 mmol/L in 2 of 22 (9.1%) in the CYTO/DIS+ group, whereas no cows in the HEALTHY or CYTO/DIS- group had BHB concentrations above 1.1 mmol/L. SCC data were unavailable for two cows. An SCC exceeding 200,000 cells/mL was observed in 11 of 49 cows (22.4%) in the HEALTHY group and 3 of 14 cows (21.4%) in the CYTO/DIS− group. In the CYTO/DIS+ group, 8 of 21 cows (38.1%) had an SCC exceeding this threshold.

### 3.2. Associations Between APP and Metabolic Marker Concentrations and Study Groups

**Figure 1:**
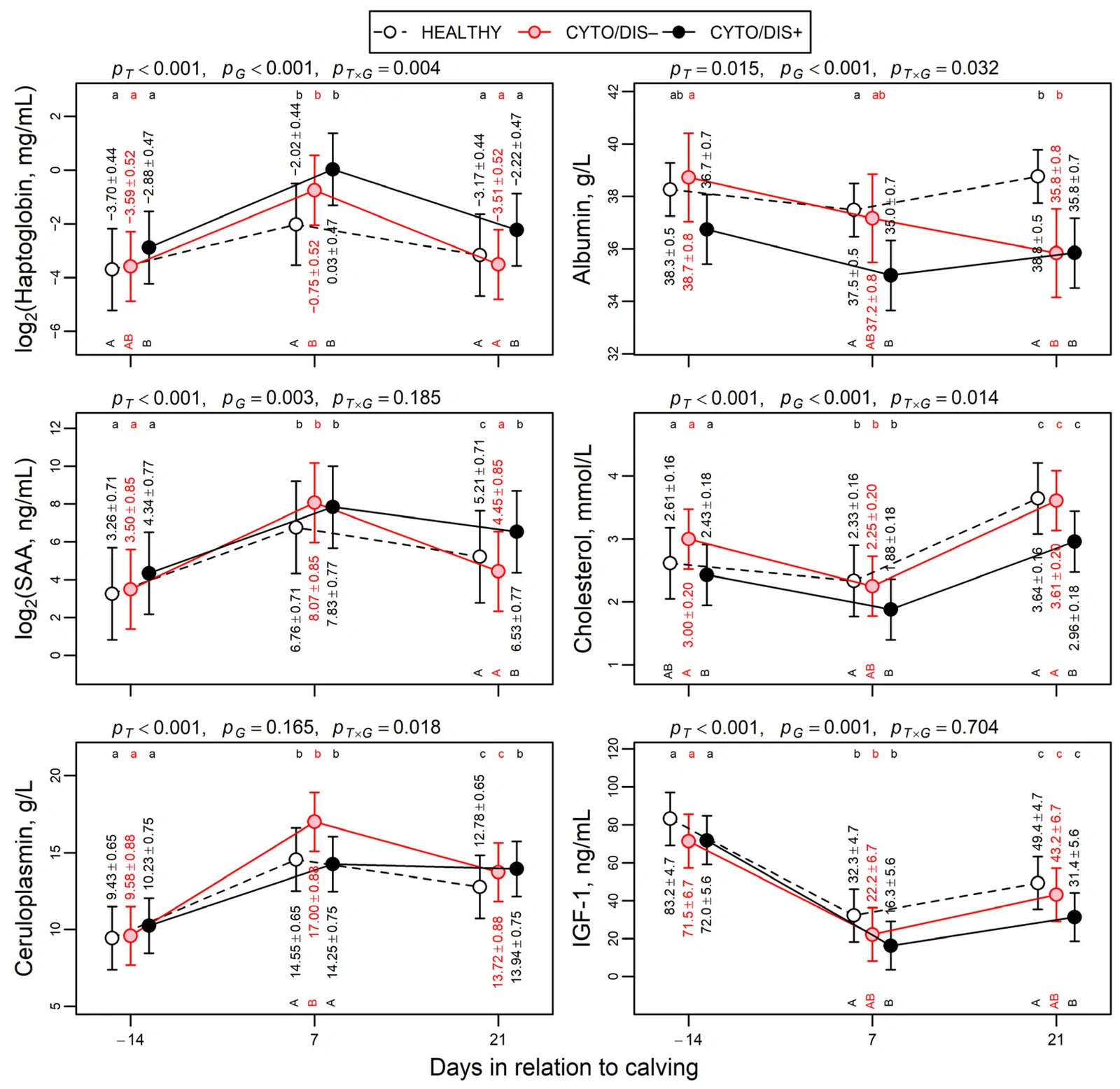
Model-based means of acute phase proteins and metabolic markers depending on group (HEALTHY, *n* = 50; CYTO/DIS−, *n* = 14; CYTO/DIS+, *n* = 22) and time period estimated with linear mixed models considering fixed effects of time, group, time by group interaction, parity (2nd vs. > 2) and body condition score before calving, and random effects of experiment and cow. Haptoglobin and serum amyloid A were analysed on a binary logarithmic scale (unit change corresponds to a two-fold difference on original scale). Error lines represent 95% confidence intervals; numerical values indicate model-based means ± standard errors. *p*-values indicate effects of time (*p_T_*), group (*p_G_*) and time by group interaction (*p_T × G_*). For each panel, means without common lowercase letters differ among timepoints within a group, and means without common uppercase letters differ among groups within a timepoint (*p* < 0.05, Tukey post hoc test). Means without letters do not differ.

Concentrations of the measured APPs and metabolic markers according to CYTO status and time, adjusted for parity and prepartum BCS, are presented in Figure 1.

Hp concentrations differed over time (*p* < 0.001), with a group effect (*p* < 0.001) and a significant interaction (*p* = 0.004). Across all measured timepoints, Hp concentrations in CYTO/DIS+ cows were higher than in HEALTHY (*p* < 0.001) and CYTO/DIS− cows (*p* = 0.006). Hp concentrations were higher in CYTO/DIS+ cows at −14 days prepartum (*p* < 0.05), at 7 (*p* < 0.001) and 21 days postpartum (*p* < 0.05) compared with HEALTHY cows. In contrast, Hp concentrations in CYTO/DIS− cows did not differ from HEALTHY cows at −14 days prepartum or at 21 days postpartum, but were higher at 7 days postpartum (*p* = 0.006). Hp concentrations at 7 days postpartum did not differ between CYTO/DIS+ and CYTO/DIS− cows.

ALB concentrations were affected by time (*p* < 0.05) and group (*p* < 0.001), with a significant interaction (*p* < 0.05). Across all measured timepoints, ALB concentrations in CYTO/DIS+ cows were lower than in HEALTHY cows (*p* < 0.001), but did not differ from CYTO/DIS− cows. ALB concentrations were lower in CYTO/DIS+ cows at 7 (*p* = 0.006) and 21 days postpartum (*p* = 0.001), and were numerically lower at −14 days prepartum (*p* = 0.141) compared with HEALTHY cows. ALB concentrations in CYTO/DIS− cows at −14 days prepartum and 7 days postpartum did not differ from HEALTHY cows, but were lower at 21 days postpartum (*p* = 0.008). At 21 days postpartum, ALB concentrations did not differ between CYTO/DIS− and CYTO/DIS+ cows.

SAA concentrations differed over time (*p* < 0.001) and among groups (*p* = 0.003), with higher concentrations in CYTO/DIS+ cows at 21 days postpartum compared with both HEALTHY and CYTO/DIS− cows (*p* ≤ 0.05 and *p* < 0.05, respectively). No significant time x group interaction was observed (*p* = 0.185).

CERULO concentrations differed over time (*p* < 0.001), with no overall group effect, although a time × group interaction was observed (*p* = 0.018). At 7 days postpartum, CERULO concentrations were higher in CYTO/DIS− cows compared with both CYTO/DIS+ and HEALTHY cows (*p* < 0.05). CERULO concentrations at 7 days postpartum did not differ between HEALTHY and CYTO/DIS+ cows.

CHOL concentrations were affected by time and group (*p* < 0.001), with a significant interaction (*p* = 0.014). Across all measured timepoints, CHOL concentrations in CYTO/DIS+ cows were lower than in HEALTHY (*p* < 0.001) and CYTO/DIS− cows (*p* = 0.001). CYTO/DIS+ cows had lower CHOL concentrations at −14 days prepartum compared with CYTO/DIS− cows (*p* < 0.05), and lower concentrations at both 7 and 21 days postpartum compared with HEALTHY cows (*p* < 0.01 and *p* < 0.001, respectively). CHOL concentrations were also lower in CYTO/DIS+ cows compared with CYTO/DIS− cows at 21 days postpartum (*p* < 0.01). CHOL concentrations in CYTO/DIS− cows did not differ from HEALTHY cows at any timepoint.

IGF-1 concentrations differed over time (*p* < 0.001) and among groups (*p* = 0.001), without a significant time x group interaction (*p* = 0.704). Across all measured timepoints, IGF-1 concentrations were lower in CYTO/DIS+ cows (*p* = 0.002), while lower concentrations were observed in CYTO/DIS- cows, although the difference was not statistically significant (*p* = 0.160), compared to HEALTHY cows. IGF-1 concentrations were lower at 7 and 21 days postpartum in CYTO/DIS+ cows compared with HEALTHY cows (*p* < 0.05 and *p* < 0.01, respectively). No differences were observed at any timepoints between CYTO/DIS− and HEALTHY cows or between CYTO/DIS− and CYTO/DIS+ cows.

## 4. Discussion

This study aimed to compare plasma APP and metabolic marker concentrations and their dynamics during the transition period in cows diagnosed with CYTO at day 40 ± 2 postpartum, with and without concurrent clinical disease or calving-related disorder, and healthy (CYTO-negative) cows.

Our findings support a role for inflammation during the transition period as a potential risk factor in the pathogenesis of CYTO. Moreover, our data are in agreement with previous reports showing that CYTO occurs in clinically healthy cows [8,23,24] and also subsequent to clinical disease [47,48], and further demonstrate that the magnitude and temporal profile of the inflammatory response differ between these two groups of cows.

Cows with clinical disease (uterine and non-uterine) that progressed to CYTO (CYTO/DIS+) had elevated prepartum Hp concentrations, potentially reflecting an underlying inflammatory response. At the measured postpartum time points, they also exhibited higher Hp and SAA concentrations and lower CHOL, ALB and IGF-1 concentrations, potentially reflecting more pronounced inflammatory and metabolic alterations. In contrast, clinically healthy cows that developed CYTO (CYTO/DIS−) appeared to experience a more transient postpartum inflammatory response, reflected by elevated Hp and CERULO at day 7 postpartum and lower ALB at day 21 postpartum.

These findings are consistent with the concept that postpartum CYTO is closely linked to systemic inflammatory status [6,7], and that impaired adaptation to the transition period contributes to uterine immune dysfunction [6,7,49,50]. In addition, the present study identifies specific APP patterns associated with CYTO development that appear to be independent of concurrent clinical disease.

Cows that subsequently developed CYTO, irrespective of clinical disease status, exhibited significantly higher Hp concentrations at day 7 postpartum and lower ALB concentrations at day 21 postpartum compared with HEALTHY cows. This observation suggests that certain APPs may reflect underlying pathophysiological processes linked to CYTO rather than simply the presence of clinical disease.

Although numerous studies have examined the association between increased early-postpartum Hp concentration and uterine diseases [27,51,52,53], only a limited number have focused on subclinical CYTO. Furthermore, their observations appear to be inconsistent. Specifically, some studies have reported a higher Hp concentration about one week postpartum in cows that developed subclinical CYTO compared to healthy cows [23,24,27], whereas other studies observed no difference at the same timepoint between healthy cows and those that developed subclinical CYTO [54,55]. In the present study, Hp concentrations were significantly higher at day 7 postpartum in CYTO-positive cows, regardless of clinical disease status (i.e., in both CYTO/DIS− and CYTO/DIS+ cows). Additionally, we also observed prepartum Hp elevation in CYTO/DIS+ cows, suggesting that some animals may enter the postpartum period with a pre-existing inflammatory response, which may be associated with subsequent clinical disease and the development of CYTO. A link between prepartum inflammation and the development of postpartum diseases has previously been described. For example, elevated Hp concentrations one week prior to parturition have been associated with an increased risk of retained placenta and metritis [28]. In addition to uterine diseases, prepartum acute phase response has also been associated with the development of lameness [30], ketosis [31] and subclinical hypocalcaemia [32].

The temporal dynamics of postpartum ALB concentrations differed among HEALTHY, CYTO/DIS+ and CYTO/DIS− cows. ALB concentrations were lower in CYTO/DIS+ cows than in CYTO/DIS− and HEALTHY cows at day 7 postpartum. However, at day 21 postpartum, ALB concentrations were lower in both groups of CYTO-positive cows (i.e., regardless of clinical disease status) compared with HEALTHY cows.

Previous studies have shown that lower ALB concentrations at day 35 postpartum are associated with subclinical CYTO [23] and that higher uterine PMN percentages at day 42 postpartum, including cows with clinical endometritis, are associated with lower ALB levels during the transition period [25].

Decreased ALB concentration is commonly interpreted as a marker of ongoing inflammation, as hepatic synthesis is downregulated during the acute phase response [14,56]. In addition, reduced ALB concentrations may reflect altered liver function and the inflammatory response [19,25,57] or negative energy balance during the metabolically demanding transition period [58,59]. Because ALB has a relatively long half-life in cattle (approximately 16.5 days) [60] and decreased concentrations can arise from multiple underlying factors, its interpretation in isolation is challenging. However, when interpreted alongside increased Hp concentrations, decreased ALB concentrations may reflect an inflammatory response. This inverse relationship between increased Hp and decreased ALB was evident in the temporal dynamics of these APPs in CYTO/DIS+ cows, which already had elevated Hp concentrations before calving and reduced ALB concentrations at day 7 postpartum. In CYTO/DIS− cows, the pattern of increased Hp and decreased ALB concentrations appeared temporally shifted, with elevated Hp concentrations observed at day 7 postpartum and decreased ALB concentrations at day 21 postpartum.

Positive APPs such as CERULO and SAA showed distinct patterns in CYTO/DIS− and CYTO/DIS+ cows. The plasma SAA concentration at day 21 postpartum was higher in CYTO/DIS+ cows in comparison with CYTO/DIS− and HEALTHY cows. Given that SAA responds rapidly to inflammatory stimuli [61], elevated SAA concentrations may reflect the cumulative inflammatory burden of clinical diseases rather than CYTO itself. Plasma CERULO concentrations were significantly higher at day 7 postpartum in CYTO/DIS− cows, whereas CYTO/DIS+ cows did not exhibit elevated levels. This finding is difficult to interpret, and any explanation remains speculative. To the best of our knowledge, no studies have evaluated CERULO in relation to the development of subclinical CYTO. Kaya et al. [26] reported increased CERULO concentrations in cows with clinical endometritis at 28–32 days postpartum, although concurrent CYTO was not assessed. While differences in sampling time may partly contribute to these inconsistencies, one possible explanation relates to CERULO synthesis, which depends on hepatic copper availability derived from dietary intake. Reduced dry matter Intake (DMI) may limit copper supply, and as a proportion (approx. 15–20%) of circulating copper is bound to ALB for transport to the liver [62], decreased ALB concentrations may further restrict its availability. Thus, the CERULO concentrations observed in CYTO/DIS+ cows with decreased ALB may reflect altered copper metabolism and reduced DMI rather than the absence of inflammatory stimulation. Indeed, CERULO activity has been shown to reflect serum or plasma copper concentrations [63,64], supporting a potential link between copper availability and CERULO synthesis. However, it has also been shown that plasma CERULO activity and copper concentration are increased at onset of lactation [64]; therefore, our interpretation remains speculative in the absence of direct measurements of copper status and DMI.

In the present study, CHOL and IGF-1 showed distinct temporal patterns. During the postpartum period, CYTO/DIS+ cows showed lower CHOL concentrations at days 7 and 21 compared with HEALTHY cows, whereas no differences were observed between CYTO/DIS− and HEALTHY cows. In dairy cows, plasma CHOL concentrations decrease during the transition period, reaching a nadir around calving, and increase gradually thereafter [18,20]. One study reported that lower postpartum CHOL concentrations were associated with severe metritis and the occurrence of multiple clinical disorders after calving, with each 0.4 mmol/L decrease in serum CHOL associated with nearly twice the likelihood of developing multiple postpartum diseases [21]. Similarly, in our study, CYTO/DIS+ cows had lower CHOL concentrations than healthy cows, supporting an association between reduced postpartum CHOL concentrations and postpartum disease occurrence. The pre-calving CHOL concentrations of both CYTO-positive cow groups did not differ from those of HEALTHY animals, but the CHOL concentration in CYTO/DIS− cows was statistically higher than in CYTO/DIS+ cows, showing differences in CHOL metabolism between these groups. High prepartum serum concentrations of CHOL have been shown to be associated with increased odds of retained placenta in a previous study [65]. Thus, prepartum high CHOL is not necessarily evidence of favourable metabolic status.

IGF-1 concentrations were reduced only in CYTO/DIS+ cows during the postpartum period. This is consistent with the established role of negative energy balance in suppressing IGF-1 synthesis [66] and with systemic inflammation-induced GH resistance [22,67]. As IGF-1 is essential for cellular proliferation, differentiation and tissue repair, reduced availability may impair postpartum uterine recovery and immune function [68,69], thereby increasing susceptibility to postpartum uterine inflammatory diseases [70], including CYTO [35]. In the present study, CHOL and IGF-1 concentrations did not differ between CYTO/DIS− and HEALTHY cows, which contrasts with previous findings reporting lower CHOL at days 14 and 21 postpartum and lower IGF-1 at day 14 postpartum in cows that developed subclinical CYTO compared with healthy cows [23]. A possible reason for these discrepancies may be a more favourable metabolic status of the CYTO/DIS− cows in the present study.

Taken together, our findings suggest that cows with CYTO and concurrent clinical disease or calving-related disorder differ from those with CYTO in the absence of clinical disease in their pre- and postpartum inflammatory and metabolic profiles. These differences may provide further insight into the systemic responses associated with concurrent clinical disease and uterine inflammation. In this context, previous findings of a higher prevalence of CYTO in clinically diseased cows than in healthy cows (43.1% vs. 20.6%, *p* = 0.009) [12] further highlight the association between clinical disease and uterine health. However, the relationships between systemic inflammatory and metabolic changes, concurrent clinical disease, and postpartum uterine inflammation remain to be fully elucidated. The associations identified in this relatively small cohort from a single herd warrant further investigation. Larger, more comprehensive studies are needed to evaluate the effects of pre- and postpartum acute phase responses, metabolic markers, concurrent diseases, and the subsequent development of CYTO.

## 5. Conclusions

The pattern of inflammatory and metabolic markers during the transition period associated with CYTO development differs according to the presence of concurrent clinical disease or calving-related disorder. Irrespective of concurrent clinical disease status, cows with CYTO had higher haptoglobin concentrations at 7 days postpartum and lower albumin concentrations at 21 days postpartum than healthy cows.

## Figures and Tables

**Table 1:** Descriptive characteristics of multiparous Holstein dairy cows in the three study groups.

Variable	HEALTHY (*n* = 50)	CYTO/DIS− (*n* = 14)	CYTO/DIS+ (*n* = 22)
Parity	2.2 ± 0.50 ^a^	2.6 ± 0.76 ^ab^	2.9 ± 1.11 ^b^
BCS before calving	3.2 ± 0.27 ^a^	3.1 ± 0.28 ^a^	3.2 ± 0.22 ^a^
45-day cumulative milk yield (kg)	1564.2 ± 227.5 ^a^	1592.0 ± 394.1 ^a^	1221.5 ± 442.0 ^b^
PMN % (40 ± 2 days postpartum)	3.1 ± 2.18 ^a^	48.8 ± 34.21 ^b^	52.1 ± 34.20 ^b^

^a,b^ For each variable, groups without a common superscript letter are statistically significantly different (*p* ≤ 0.05, pairwise Wilcoxon test followed by multiple testing correction with FDR method). Data represent mean ± SD.

**Table 2:** Calving-related disorders and clinical diseases diagnosed in the 22 cows in the CYTO/DIS+ group.

Calving-Related Disorder or Clinical Disease	Number of Cows
**Cows with a single disorder or disease**	
Twin calving	1
Clinical hypocalcaemia	2
Metritis	4
Purulent vaginal discharge	2
Clinical mastitis	1
Severe lameness	3
**Cows with two or more disorders or diseases**	
Twin calving and metritis	2
Twin calving, dystocia and metritis	1
Retained placenta and metritis	1
Metritis and purulent vaginal discharge	1
Retained placenta and purulent vaginal discharge	2
Retained placenta, clinical hypocalcaemia and purulent vaginal discharge	1
Twin calving, dystocia and severe lameness	1
**Total**	**22**

## Data Availability

The data that support the findings of this study are available on reasonable request from the senior author.

## Abbreviations

The following abbreviations are used in this manuscript:

ALB	albumin
APPs	acute phase proteins
BCS	body condition score
BHB	β-hydroxybutyrate
CERULO	ceruloplasmin
CHOL	cholesterol
CYTO	cytological endometritis
CYTO/DIS+	cytological endometritis with clinical disease
CYTO/DIS−	cytological endometritis without clinical disease
DMI	dry matter intake
GH	growth hormone
Hp	haptoglobin
IGF-1	insulin-like growth factor-1
PMNs	polymorphonuclear neutrophils
SAA	serum amyloid A
SCC	somatic cell count
